# Supplementary material for: Content-rich biological network constructed by mining PubMed abstracts
Source: BMC Bioinformatics. 2004 Oct 8;5:147. doi: 10.1186/1471-2105-5-147 (PMC528731; doi:10.1186/1471-2105-5-147)
Supplement: Additional File 2 — The original results of the above study (non-essential files are deleted to keep the file size under the limit set by BMC bioinformatics). [file 1471-2105-5-147-S2.bz2 › chilibotAdditionalFile2/dip05/160ID8662691E563/html/SLC2A1_CALR.html]

 


 **SLC2A1** and **CALR** 
  
Found 1 abstracts in PubMed, retrieved 1.  
 

 What does Google say? 
 PDF only 
| .edu only 

---

**Interactive relationship** (e.g. stimulation, inhibition, etc)

**Neutral relationship**- The glut 1  [ **SLC2A1** ]  glucose transporter interacts with calnexin and calreticulin  [ **CALR** ] .  Ref: 8662691 J Biol Chem, 1996

**Non-interactive relationship** (e.g. studied together, co-existance, homology, etc.)

- Thus, our data show that both calnexin and calreticulin  [ **CALR** ]  with Glut 1  [ **SLC2A1** ]  in a glycosylation dependent manner.  Ref: 8662691 J Biol Chem, 1996
